# A New Look at Cultivar Preference in *Hoplocampa testudinea* (Hymenoptera: Tenthredinidae) on Apple in the Annapolis Valley of Nova Scotia, Canada

**DOI:** 10.3390/insects12090769

**Published:** 2021-08-27

**Authors:** Suzanne Blatt, Kim Hiltz

**Affiliations:** Agriculture and Agri-Food Canada, 32 Main Street, Kentville, NS B4N 1J5, Canada; kim.hiltz@agr.gc.ca

**Keywords:** *Hoplocampa testutindea*, cultivar preference, preference-performance hypothesis, European apple sawfly

## Abstract

**Simple Summary:**

Many insect species show a preference for specific varieties or cultivars within a host plant type, e.g., apple. The European apple sawfly, *Hoplocampa testudinea* Klug was found to show preference for apple cultivars in Nova Scotia in 2013 and 2014. We hypothesized that this preference could result from either the female selecting specific cultivars for egg deposition or differential survival of the larvae on these cultivars. We studied 15 cultivars over a four-year period (2016–2019) to determine the distribution of egg deposition within the orchard, we bagged fruitlets to closely monitor the damage and impact of *H. testudinea* during the growing season and evaluated the fruitlets for soluble solids (sugars), acidity and firmness. We determined that female choice in combination with fruitlet chemistry is likely responsible for the cultivar preferences observed.

**Abstract:**

(1) Background: The European apple sawfly, *Hoplocampa testudinea* Klug (Hymenoptera: Tenthredinidae), can be an economically important pest in eastern Canada and shows preference for apple cultivars in Nova Scotia, Canada. We hypothesized that this preference could be due to oviposition by female *H. testudinea* (preference-performance hypothesis) during the bloom period or differential larval survival during development due to fruitlet physicochemical properties. (2) Methods: Fifteen commercial and experimental apple (*Malus* *domestica* Borkh.; Rosaceae) cultivars located at the Kentville Research and Development Centre (Kentville, Nova Scotia) were chosen and examined for *H. testudinea* oviposition, larval performance during fruitlet development, fruitlet physicochemical properties and damage assessment at harvest from 2016–2019, inclusive. (3) Results: *H. testudinea* showed significant cultivar preference during oviposition, during development and at harvest, but the ranking of these cultivars was not the same throughout the season. Total impact by *H. testudinea* was consistent for most cultivars over multiple years of the study. (4) Conclusion: Correlation of oviposition with damage provided weak evidence for the preference-performance hypothesis. We propose that this relationship is weak due to differential survival of larvae during development.

## 1. Introduction

Preference of insects for specific host genotypes has been broadly observed across numerous agricultural crops and insect families [1] and references therein. Identifying and understanding the role of host genotypes showing resistance to insect damage is desired for the purpose of enhancing pest management programs [2,3,4,5]. For pests that occur at times throughout the production cycle when pesticide applications would be detrimental to beneficial predators and pollinators, use of resistant genotypes (cultivars or varieties) offers a control option that is effective and able to conserve naturally occurring ecosystem services. One such pest is the European apple sawfly, *Hoplocampa testudinea* (Klug) (Hymenoptera: Tenthredinidae), which emerges and oviposits during the bloom period of apple (*Malus domestica*, Borkh., Rosaceae).

A recent review by Vincent [6] describes the distribution and introduction of the European apple sawfly into North America and Canada. In brief, *H. testudinea* was reported on Vancouver Island, Victoria British Columbia in 1940 [7,8]. Over the next several decades it spread into the western and northeastern United States of America then north into British Columbia and Québec, Canada and finally eastward into the Maritimes [9,10,11,12,13]. Damage from *H. testudinea* occurs post-mating when the female will lay an egg at the base of the apple blossom [11,14,15]. Larvae require 1–2 weeks to develop [11,16] and upon hatching burrow into the fruitlet. As larvae grow, they leave the first fruitlet and invade nearby fruitlets, consuming one fruitlet per instar [11,17]. After the final instar, larvae leave the fruitlet and drop to the ground where they overwinter as pupae in the soil. Adult female *H. testudinea* emerge from the soil in early to mid-May [14] and mature within 4–10 days [8,11,15,16,18].

Cultivar preference in *H. testudinea* is not a novel idea. Briggs and Alston [19], Alford [20], Hogmire and Miller [21] and Burgart [22] all suggested that *H. testudinea* prefers certain cultivars, based upon observed damage assessments conducted at harvest. Burgart [22] found preferred cultivars early in the season (based upon adult visitation during bloom, when oviposition is occurring) to be different from the ranking observed during fruitlet development (based upon visual surveys of infested fruitlets) and again different from the ranking observed at harvest (based on observed damage). As the cultivars showing high levels of *H. testudinea* damage differed throughout fruit development, this suggests three things: 1. that female *H. testudinea* could be preferentially selecting cultivars in the spring for oviposition; 2. that larvae could be experiencing differential mortality across cultivar (antibiosis); and 3. that evaluating cultivar preference in the fall based on primary damage alone may not accurately reflect cultivar susceptibility or the full impact of *H. testudinea* on apple production. We endeavored to evaluate these hypotheses through closer investigation of the life cycle of *H. testudinea* in connection with its host, *M. domestica*.

During the development, *Hoplocampa testudinea* can cause two types of damage: primary and secondary. Primary damage is caused following egg eclosion when the larva burrows into the developing fruitlet. This feeding occurs just below the skin and results in a thin line which turns purple and has a distinctive “c” shape, referred to as a “c-scar” [16,23]. Secondary damage occurs when the larva has grown to second (or later instars) and requires another fruitlet to continue development. The exit hole from the first fruitlet and entrance hole into the side of the second and subsequent fruitlets, often filled with frass, is readily observed in late June and early July. Fruitlets with secondary damage drop, or are aborted, from the tree during mid-July and are not observed at harvest. Fruitlets with primary damage only (no exit hole) will remain on the tree and continue to develop. From mid-summer onwards into harvest, these apples exhibit the characteristic c-scar [16,23] and are used to evaluate the extent of damage by *H. testudinea*. Fruitlets with primary damage only suggest a failure of the larvae to survive past the first instar.

Typically, percent damage and cultivar preference of *H. testudinea* are evaluated at harvest based solely on observed primary damage. If larval survival is differentially affected by cultivar, this percentage would only represent those larvae which did not survive to leave the first fruitlet and does not include those fruitlets which dropped from the tree with either primary or secondary damage. The full impact of *H. testudinea* is the combination of those fruits with primary damage remaining on the tree until harvest and fruitlets with secondary damage that have fallen off earlier in the growing season. The influence of cultivar on the development of *H. testudinea* larvae has not been examined nor considered in previous assessments of cultivar preference. As such, a high frequency of primary damage at harvest may indicate cultivar tolerance to *H. testudinea*, rather than preference [19,20]. The association between fruit quality and pest incidence has been documented in apple for some pests [24]. Whether fruitlet quality, as evaluated using certain physicochemical properties, could be influencing the development of *H. testudinea* is unknown. The observation of certain genotypes being preferentially selected by insects can be explained by the preference-performance hypothesis where females select hosts that should provide the best environment for their offspring [25,26]. In the case of *H. testudinea*, this would result in an observed difference in oviposition across apple cultivars and a high correlation between number of eggs or percentage of clusters with eggs and resulting damage (i.e., secondary damage or total damage). If oviposition across the cultivars is equal, then differential survival of the larvae during development may suggest antibiosis [27] as shown in *Castanea sativa* where variety leads to differential gall development and survival of the larvae [28]. In apples, antibiosis has been documented for a mite [27] but not *H. testudinea*.

The objectives of this work were to: (1) examine female oviposition preference across cultivars, (2) assess larval performance across cultivars during fruitlet development, (3) elucidate the full impact of *H. testudinea* across apple cultivars and (4) elucidate any relationships between select fruitlet physicochemical properties and *H. testudinea* primary and secondary damage.

## 2. Materials and Methods

### 2.1. Orchard Blocks

Entomological research apple blocks contain experimental and commercial cultivars and are located at the Kentville Research and Development Center (KRDC) in Kentville, Nova Scotia, Canada (45°04′08″ N, 64°28′41″ W). The blocks were established in 1999 and 2000, B137 and B138, respectively, and consist of cultivars grafted onto commercially available Malling-9 rootstock (B137) and Budagovski-9 rootstock (B138). Trees were topped at 3.5 m in height, and managed organically from 1999 through 2011, then treated with fungicides to control apple scab, but not with any insecticidal sprays since 2012. Each block is comprised of two rows of trees, spaced 1.5 m apart, with cultivars randomized within the block and allocated equally to each row. Each cultivar in B137 (20 in total) had five replicate trees and each cultivar in B138 (13 in total) had eight replicate trees. Distance between the blocks was approximately 30 m with the B137 block located north of the B138 block. Rows were in line between the blocks. These blocks were surrounded by a road on the north, fields of winter wheat on the east and south sides and a mixed cultivar block of apple to the west.

### 2.2. Damage Assessments: 2010–2014 and 2015–2019

From 1999 through 2014 inclusive, 27 apples from each tree within each cultivar were assessed for insect damage at harvest. *H. testudinea* damage was first observed in these blocks in 2009 but only on 2 cultivars (“Chinook” in B137 and B138 and “s14-15-72” in B138, data not shown). It is unknown when *H. testudinea* was first documented in Nova Scotia, and this observation represents the first record of damage from this pest at this site. By 2010, damage was distributed throughout both blocks and we chose to use this year as the start of the sampling period (2010–2014). From 2015–2017 inclusive, 27 apples from each tree were taken from all trees across a subset of cultivars (15 in total) within each block, eight from B137 and seven from B138 with no overlapping cultivars between blocks (see Table 1) and assessed for damage from *H.* *testudinea*. Due to a hurricane in 2018 and a June freeze in 2019 there were insufficient apples on many of the trees to collect this number independent of the bagging study, so we used the damage observed in the bagging study (range of four to 27 apples/tree in 2018 and seven to 40 apples/tree in 2019). For the oviposition preference and fruitlet assessments the target was 5 replicate trees from each of the cultivars listed in Table 1. In any given year for any given cultivar there may not have been adequate fruit on a tree or there may not have been at least three trees within the cultivar, due to biennial bearing or environmental challenges, e.g., an unexpected freeze in June 2018.

### 2.3. Oviposition Preference

To evaluate if adult female *H. testudinea* are preferentially laying eggs on certain cultivars, flower clusters were examined for eggs in mid-June (at petal fall) in 2016 through 2019 inclusive. Nine or 10 flower clusters were collected from four to five trees from a subset of cultivars (see Table 2) and brought to the lab for assessment. Each fruitlet within the cluster was dissected with a razor blade under a stereomicroscope. Number of *H. testudinea* eggs present on the king bloom and number of eggs present on the lateral flowers were counted.

### 2.4. Bagging Study

*Fruitlet assessments*—to examine the impact of *H. testudinea* on fruitlet development, clusters from each tree used in the oviposition preference study were bagged using pollination bags (DelStar Technologies, Inc., Austin, TX, USA) measuring 30 cm × 45 cm. Clusters were chosen at random and needed to have at least 3 fruitlets within the cluster. Pollination bags were put on trees when the fruitlets reached an average size of 10 mm diameter. In 2016, our target was 20 clusters/tree to be bagged, in 2018 and 2019 this was reduced to 10 clusters/tree as fewer bags than expected were lost (blown from the trees or fruiting spur snapped off) in 2016. Bags were removed and fruitlets assessed for *H. testudinea* damage in late July or early August. Actual number of bags that on each variety are reported in Supplemental Table S1. The variation in these numbers is due to some varieties and trees exhibiting biennialism and thus having few or no fruitlets available for the study in any given year and for 2018 our study was impacted by a hurricane which removed many of the bags prior to their intended removal date. Fruitlets were characterized as having either primary or secondary damage, were healthy, damaged by other insects or failed to develop (low pollination).

*Fruitlet chemistry*—it was hypothesized that chemical characteristics of the fruitlets could correlate with observed damage if these were influencing larval survival and/or development. Using the same cultivars and trees within each year as used for the Oviposition and Bagging studies, the initial assessment of fruitlet chemistry in 2016 occurred in late July, just ahead of bag removal and damage assessment, when the fruitlets measured approximately 30 mm in size. Twenty (20) undamaged fruitlets per tree were sampled and brought to the lab for chemical assessment of skin thickness and flesh firmness (pressure required to break the skin and penetrate the flesh), soluble solids (Brix) and titratable acidity. Pressure was determined using a penetrometer (Fruit Quality Tester, Geo-Met Instruments, Inc., New Minas, NS, Canada) on each fruitlet and recorded as foot pounds (ft lb) of force. A pooled juice sample from the fruitlets was used to determine percent soluble solids (Brix) using a refractometer (PAL-1, Atago Co. Ltd., Minato-ku, Tokyo, Japan) and acidity was determined by titration using 1.0 N NaOH (865 Dosimat Plus, Methohm AG, Herisau, Switzerland) with 1 mL of juice diluted into 50 mL of reverse osmosis water. To evaluate any change in fruitlet chemistry during development, follow-up studies were conducted in 2017, 2018 and 2019. Twenty (20) fruitlets from the same cultivars (and trees within each year) as used in the oviposition study were collected when the fruitlets were 7–10 mm in diameter, at 15–25 mm in diameter and at 30–40 mm diameter. These sizes were selected to correspond with the time of bagging, halfway through *H. testudinea* larval development and when *H. testudinea* would have left the fruitlets and fallen to the ground to pupate.

### 2.5. Data Analyses

For each analysis, diagnostic statistics were used to evaluate homogeneity of variance (Levene test) and normality of the residuals (Shapiro-Wilks test) using rstatix in R prior to use of any transformation. When data did not meet the assumptions of homogeneity or normality, data were transformed using sin^−1^(x + 0.1) (if proportional) or log(x + 1) (if counts) prior to analysis.

*Damage data*—2010–2014 data were analyzed using a linear mixed effects model (lmer in the lme4 package) in R software [29], with “cultivar” as a fixed variable and replicate (tree) nested within cultivar within year. Percentage of primary damage from *H. testudinea* was transformed using (sin^−1^ + 0.1) then analyzed with cultivar included in the model and not included in the model. The two models were compared using anova to determine the significance of cultivar. As some cultivars overlapped between B137 and B138, analysis was conducted on each block separately. Damage data from 2015–2019 were analyzed using a linear mixed effects model (lmer in the lme4 package in R) with year as a random variable. With the reduced number of replicates within each cultivar we were unable to nest replicate (tree) within cultivar as we did for the 2010–2014 data set. As cultivars were not repeated across the blocks, this variable was not included in the model and all cultivars compared using pair-wise contrasts using emmeans (emmeans package in R).

*Oviposition study*: number of eggs oviposited on flower clusters, percentage of clusters with eggs, percentage of eggs on the King fruit and on the lateral fruitlets were analyzed for differences between cultivar using anova (aov) methods in R for each year separately, then for all years with year included in a linear mixed model anova as a random variable using the lme4 package.

*Bagging study*—*fruitlet assessments*: mean percentage of fruitlets showing secondary damage (dropped from the tree), primary damage (dropped from the tree), primary damage (still attached and developing), healthy, damaged by other insects or failed to develop were compared between cultivar using anova (aov) methods in R for each year separately, then for all years with year included in a mixed model anova as a random variable using the lme4 package.

*Bagging study*—*fruitlet chemistry*: for the data collected in 2017–2019 inclusive and at each fruitlet size, each variable (soluble solids, acidity and pressure) was compared between cultivars using anova methods in R for each year separately, then for all years using a mixed model anova with year as a random variable using the lme4 package.

For all analyses where cultivar was significant, differences were evaluated using a post-hoc Tukey’s HSD (honestly significant difference), using the agricolae package in R. Linear regression (lm in R) was used to examine the relationships between eggs per cluster and damage by *H. testudinea* (primary, secondary or total), and between damage by *H. testudinea* and fruitlet characteristics using data from 2016, 2018 and 2019 (as no bagging study occurred in 2017).

## 3. Results

### 3.1. Damage Assessment at Harvest

At harvest, *H. testudinea* primary damage is observed as a “c” shaped scar. Data from 2010–2014 showed significant cultivar preference by *H. testudinea* (Table 1) in each block (B137: χ^2^ = 100.9, *p* < 0.0001, B138: χ^2^ = 72.9, *p* < 0.0001). Within B137, damage ranged from 2%–12% and, within B138, from 3%–12%. Cultivars with the greatest damage were “Chinook” (~12% in both B137 and B138), “S14-15-72” (~12% in B138), “NJ 90” (~11% in B137) and “NY-79-507-72” (~11% in B137). Cultivars with the least amount of damage were “Ambrosia” (~2%), “COOP 29” (~3%), “NJ 109” (~2%) and “Zestar!” (~2%) in B137 and “s47-23-100” (~3%) and “S23-06-153” (~5%) in B138. Cultivars in B138 had higher levels of damage than cultivars in B137.

Data from 2015–2019 on a subset of these cultivars found percentage of fruit on the tree at harvest with *H. testudinea* damage to be significant across cultivar (F_14,224_ = 2.07, *p* < 0.01), Table 1. Cultivars within B137 differed significantly for percentage of apples with *H. testudinea* damage at harvest (F_7,114_ = 2.11, *p* = 0.04) but not in B138 (F_6,110_ = 1.74, *p* = 0.11). Comparing across blocks, “Chinook” in B138 had the highest level of such damage (~9%) while “Zestar!” in B137 had the least (~2%). Comparing the subset of cultivars (2015–2019) with damage from *H. testudinea* with damage on the same cultivars from 2010–2014, shows more than 50% of these cultivars to exhibit a similar level of damage in both sampling periods., e.g., “Summerland McIntosh” had approximately 5% of apples showing damage across 2015–2019, as well as across 2010–2014. Cultivars showing greater than 1% difference between these two sampling periods include “COOP 39”, “Hampshire”, “Jubilee Fugi”, “NJ 109”, “8S-27-43”, “S14-15-72”. Ranking of the subset of cultivars based upon damage observed at harvest in both sampling periods shows cultivars “NY79-507-72” and “COOP 39” to be in the top three for damage and “Zestar!” to have the lowest damage in B137. “Delblush” showed similar damage across both sampling periods while “Jubilee Fugi” and “NJ 109” changed their position the most, moving from high damage (early sampling period) to less damage (later sampling period) and from low damage (early sampling period) to higher damage (later sampling period), respectively. Cultivars in B138 had “Chinook” and “S14-15-72” showing the highest damage across both sampling periods, with “S47-23-100” and “Summerland McIntosh” showing low damage in both sampling periods. Cultivar showing the greatest change in ranking was “8S-27-43” moving from high damage (8% in the early sampling period) to lower damage (3% in the later sampling period). “8S-69-23” and “Royal Gala” changed ranking over the two sampling periods but showed similar levels of damage (Table 1).

### 3.2. Oviposition Preference

In general, percentage of clusters with *H. testudinea* eggs ranged from 20%–53% across the years of the study, 2016–2019, Figure 1A. Number of clusters sampled each year ranged from 598 to 699, yet the percentage of clusters with eggs showed a significant decrease over the years of this study (*p* < 0.0001). Clusters with eggs located only on the lateral flowers varied significantly across years (*p* < 0.0001) and ranged from 11%–28%, while 5%–10% of the clusters had eggs deposited on only the King flower which did not differ significantly across years (*p* = 0.15). Percentage of clusters with eggs on both lateral and King flowers varied significantly (*p* < 0.0001) across years and ranged from 3%–15%. Cultivar was significant for percentage of clusters with eggs (*p* = 0.006) and showed significant differences both within and across years (Table 2). Across all years, percentage of clusters with eggs varied significantly across cultivar, ranging from 19.9–52.4% in B137 and 25.9%–48.6% in B138. Within each year, percentages were significant across cultivar in 2017, 2018 and 2019, but not 2016 (Table 2).

**Figure 1:**
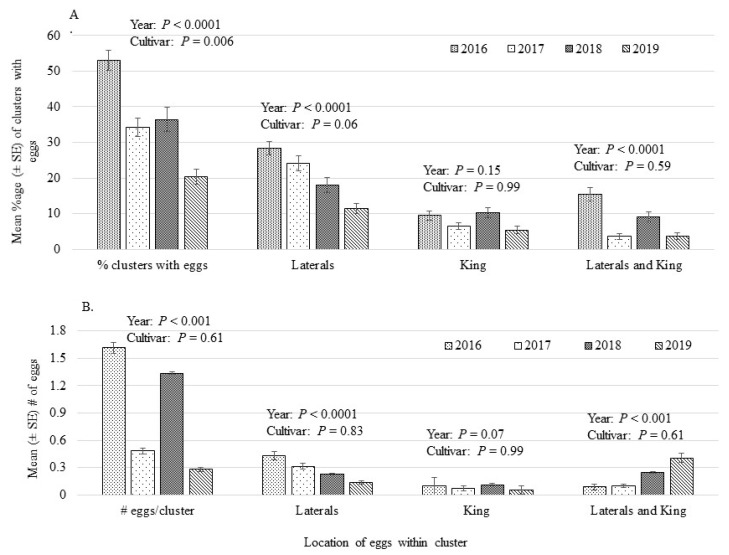
Within cluster location of eggs oviposited by *Hoplocampa testudinea* as (**A**) mean percentage of clusters and (**B**) mean number of eggs. Clusters taken from fifteen cultivars of apple with two blocks from 2016–2019, inclusive. Number of clusters examined each year: 2016—699, 2017—689, 2018—598, 2019—671. For A: F_2,79_ values (Year) and F_14,79_ (Cultivar) for % clusters with eggs: 12.72 and 2.44, Laterals: 7.36 and 1.73, King: 1.95 and 0.25, and Laterals and King: 17.25 and 0.87. For B: F_2.79_ values (Year) and F_14.79_ (Cultivar) for # eggs/cluster: 8.96 and 0.86, Laterals: 10.12 and 0.63, King: 2.64, 0.39 and Laterals and King: 8.87 and 0.86.

Number of eggs oviposited by *H. testudinea* on clusters was significant across year (*p* < 0.001) but not cultivar (*p* = 0.61, Figure 1B). Number of eggs per cluster varied by location, where clusters with eggs deposited on the laterals or laterals and King flowers being significant across study years (*p* < 0.0001 and 0.001, laterals, laterals and King, respectively). Number of eggs on the King flower did not vary across year (*p* = 0.07). Analysis of total eggs per cluster within each study year found significant differences across cultivar (*p* = 0.02, Table 3). Eggs per cluster ranged from 0.83–1.55 and although significant, Tukeys’ HSD separation test could not separate the means. Looking at each year, egg deposition in 2016 and 2019 showed significant differences between cultivar (*p* < 0.05) but not block (*p* > 0.05) with no significant differences across cultivar or block in 2017 and 2018 (*p* > 0.05). In 2017 and 2018, number of eggs oviposited per cluster were lower than in 2016 (2016: 1.00–2.21 eggs/cluster, 2017: 0.80–1.68 eggs/cluster, 2018: 0.5–1.76). Eggs per cluster varied the most in 2019 (0.2–1.81 eggs/cluster). Ranking of the cultivars based upon oviposition within each year varied, with some cultivars changing very little in their ranking, e.g., in B137 “NY79-507-72” was ranked 1st, 3rd, 2nd and 2nd out of 8 cultivars over 2016, 2017, 2018 and 2019, respectively, while other cultivars changed their ranking over the years, e.g., in B138 “Royal Gala” was ranked 2nd, 5th, 3rd and 4th out of 7 cultivars in 2016, 2017, 2018 and 2019, respectively.

### 3.3. Bagging Study

#### 3.3.1. Fruitlet Assessments

**Figure 2:**
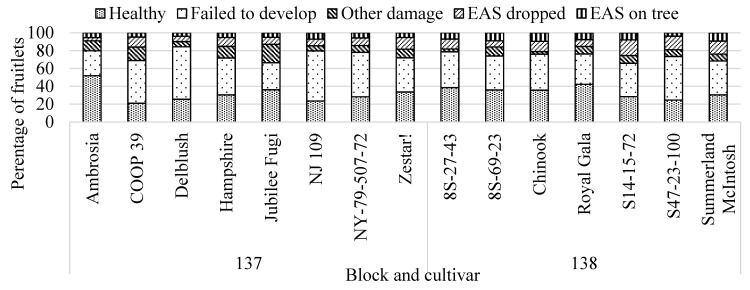
Categories of fruitlets observed in August (from 2016, 2018 and 2019) following bagging at petal-fall showing differences across cultivars from 2 blocks located at the Kentville Research and Development Centre. Percentage of fruitlets which became fruit (Healthy), did not develop (Failed to develop), were damaged by other pests (Other damage), had secondary damage from European Apple Sawfly (EAS), *Hoplocampa testudinea*, but dropped from the tree (EAS dropped) and had primary damage and remained on the tree (EAS on tree).

Across all years of the bagging study, cultivars varied significantly for the percentage of fruitlets which were healthy (F_15,78_ = 4.38, *p* < 0.0001), failed to develop (F_15,78_ = 5.65, *p* < 0.0001), and showing other damage from other insects, e.g., speckled green fruitworm *Orthosia hibisci* (F_15,78_ = 2.66, *p* < 0.002), Figure 2. Fruitlets with primary *H. testudinea* damage (still attached to the tree and continuing to develop) ranged from 3.7%–9.5% across cultivar, but this was not significant (F_15,78_ = 1.10, *p* = 0.37). Percentage of fruitlets with secondary damage from *H. testudinea* (which dropped from the tree) ranged from 3.5%–17.5% across cultivar, which was significant (F_15,78_ = 2.77, *p* = 0.001). Combining both categories of *H. testudinea* damage (primary and secondary) was also significant across cultivar (F_15,78_ = 2.85, *p* = 0.001). The ranking of cultivars for each category was not consistent, i.e., “S14-15-72” had the highest percentage (17.5%) of fruitlets with secondary *H. testudinea* damage, while “Chinook” had the highest percentage (10%) of fruitlets with primary damage. “Ambrosia” had the highest percentage of fruitlets that were healthy and become marketable fruit (51.8%) while “COOP 39” had the lowest (20.9%). Fruitlets that failed to develop (natural abortion due to low pollination) varied across cultivar and ranged from 27.9% (“Ambrosia”) to 49.8% (“NY-79-507-72”). Within each year, cultivar was significant for percentage of fruitlets which were healthy, failed to develop and other damage, but did not show significant differences across cultivar for *H. testudinea* secondary or primary damage in 2018 and 2019 (Supplementary Table S1).

#### 3.3.2. Fruitlet Chemistry

**Figure 3:**
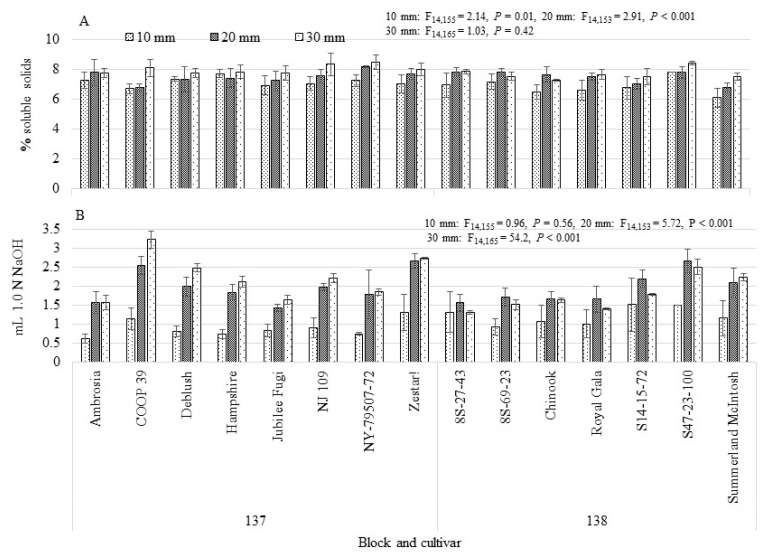
Apple fruitlets, at 10, 20 and 30 mm diameter, collected from 15 cultivars located at the Kentville Research and Development Centre in 2017–2019 and measured for (**A**) mean (±SE) percent soluble solids (Brix) and (**B**) mean (±SE) mL of 1.0 N NaOH to neutralize 1 mL of juice. Cultivar analyzed for significance within each fruitlet size category.

Across the years, percent soluble solids (Brix values) ranged from 5%–8% across cultivars with low variation between trees within cultivar (Figure 3A). When fruitlets measured 10 and 20 mm, differences between cultivars was significant (*p* = 0.01 and <0.001, 10 mm and 20 mm, respectively), but not when fruitlets reached 30 mm (*p* = 0.42). For each cultivar, as fruitlets increased in size, percent soluble solids changed significantly (F_14,112_ = 2.45, *p* = 0.005 and F_14,119_ = 2.35, *p* = 0.006, 10–20 mm and 20–30 mm, respectively) but by varying amounts (Supplemental Figure S1A). During the first stage of development (from 10–20 mm) fruitlets in all cultivars increased their soluble solids with “Royal Gala” showing the greatest increase (2%) and “COOP 39” showing the least increase (0.01%). Later in their development (20–30 mm), fruitlets within some cultivars showed a >1% increase (“COOP 39”, “Hampshire”, “NJ 109”, “S14-15-72”) while others showed a lesser increase in soluble solids (“Jubilee Fugi”, “NY-79-507-72”, “Zestar!”, “8S-27-43”, “Royal Gala”, “Summerland McIntosh”) and others showed a decrease in soluble solids (“Ambrosia”, “8S-69-23” and “Chinook”).

Acidity of fruitlets across cultivar (and across years) varied significantly with development stage (*p* = 0.56, <0.001 and <0.001, at 10, 20 and 30 mm, respectively, Figure 3B). Acidity ranged from 0.61–1.51 mL NaOH when fruitlets were 10 mm in size, from 1.42–2.66 mL NaOH at 20 mm and from 1.29–3.23 mL NaOH at 30 mm. Overall, “COOP 39” fruitlets had the most acid of the cultivars and “Ambrosia” fruitlets had the least. Change in acidity showed a similar pattern during development as the soluble solids (Supplemental Figure S1B). From 10–20 mm, the increase in fruitlet acidity was not significant across cultivar (F_14,111_ = 1.75, *p* = 0.06) and ranged from 0.60 to 1.47 mL NaOH. During the later stage of development, acidity varied significantly across cultivar (F_14,119_ = 3.31, *p* < 0.001). Some cultivars showed an increase in acidity (“Ambrosia”, “COOP 39”, “Hampshire”, “Jubilee Fugi”, “NJ 109”, “NY-79-507-72” and “Summerland McIntosh”) while the remainder showed a decrease (“Zestar!”, “8S-27-43”, “8S-69-23”, “Chinook”, “Royal Gala”, “S14-15-72” and “S47-23-100”). Cultivars which showed a decrease in both soluble solids and acidity during later development were “8S-69-23” and “Chinook”. While these increases and decreases during later development may not show statistical significance, these differences could be important for larval development.

Firmness of the fruitlets could not be measured when they were 10 mm in size as the probe was too large to obtain a reliable measurement. Fruitlets showed a steady decrease in firmness during development (Supplemental Figure S2A) for all cultivars but one, “Chinook”, which showed an increase. Firmness differed significantly across cultivar (*p* < 0.0001), with “NY-79-507-72” being the firmest cultivar and “Zestar” being the softest.

### 3.4. Correlations

The percentage of cluster with eggs showed a positive and significant (*p* < 0.0001) correlation with total (primary and secondary damage combined) and secondary damage but not primary damage (*p* = 0.43), Table 4. A positive and significant relationship was observed for total and secondary damage and mean total eggs per cluster and percentage of eggs/cluster where eggs were on both King and lateral fruitlets (*p* < 0.001) but not primary damage (*p* > 0.05). Clusters where eggs were located on only the laterals did not show a significant correlation with *H. testudinea* damage (primary, secondary or total) while eggs located on the King showed a significant correlation even though there was poor fit of the data (R^2^ = 0.09).

**Figure 4:**
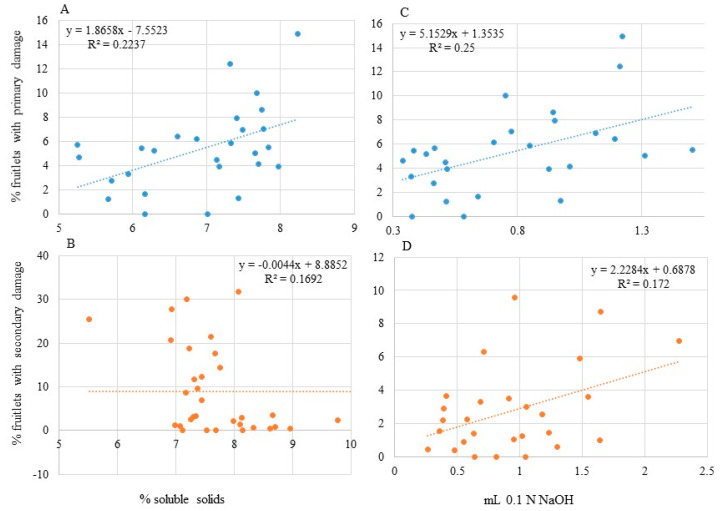
Mean percentage of fruitlets showing: (**A**) primary damage as correlated with soluble solids from fruitlets 10 mm in size, (**B**) secondary damage as correlated with soluble solids from fruitlets 30 mm in size, (**C**) primary damage as correlated with mL of 0.1 N NaOH (acidity) from fruitlets 10 mm in size and (**D**) secondary damage as correlated with change in acidity of fruitlets during development from 10 to 20 mm in size.

Fruitlet chemistry showed some significant correlation with observed *Hoplocampa testudinea* damage. Soluble solids early in development, when fruitlets were 10 mm in size, correlated with observed primary damage (*p* = 0.01, Figure 4A), while secondary damage correlated with soluble solid levels observed later in development (at 30 mm fruitlet size, *p* = 0.002, Figure 4B). Acidity of fruitlets early in development was significantly correlated with primary damage (*p* = 0.008, Figure 4C). Secondary damage was significantly correlated (*p* = 0.03) to the change in acidity as the fruitlets developed (from 10 to 20 mm in size, Figure 4D).

## 4. Discussion

There are four main results from our study: 1. cultivar preference in *H. testudinea* is consistent for many cultivars across years, 2. evaluation of cultivar choice based upon observed damage at harvest does not reflect the full impact of *Hoplocampa testudinea* in apple, 3. choice of cultivar by female *H. testudinea* is weakly correlated with total damage and 4. fruitlet chemistry weakly correlates with larval success during development.

Cultivar preference has been documented in numerous insect species over many host plants [5,30,31,32] and references therein. The potential to capitalize upon this phenomenon by selectively using resistant cultivars is well studied but with variable results [3,33,34,35,36,37,38]. Results from our multiple year study have found cultivar preference in *H. testudinea* to be fairly consistent for most of the cultivars studied. The first observation of *H. testudinea* damage in these blocks occurred in 2009 and this study has followed the increase in population, and number of cultivars impacted, over the ensuing 10 years. Of interest is the observation that, over time, some cultivars became increasingly preferred by *H. testudinea* while others became less preferred, suggesting that cues attractive to the female or fruitlet physicochemical properties can change over time. For perennial crops that can be harvested for up to or more than a decade, cultivar preference may need to be evaluated throughout their entire cropping cycle to capture both environmental conditions and physiological changes within the plant, which could alter the cues impacting host selection [39], as well as nutritive status [40].

Determination of which cultivars are preferred by *H. testudinea* is often based upon observed damage at harvest. In crops where the indicator of pest impact (i.e., bulb, leaves, stem) remains reasonably intact throughout the season, or where plant loss itself is the indicator, this is a reasonable practice. In apples, where damaged fruitlets will drop from the tree partway through the season, cultivars deemed “susceptible” based upon damage observed at harvest will underestimate the full impact of *H. testudinea*. Further, the ranking of cultivars more susceptible to impact from *H. testudinea* requires the study of the secondary damage (of fruitlets no longer on the tree) and not just evaluation of the primary damage observed at harvest. Secondary damage is indicative of larval success while primary damage, fruitlets without an exit hole, indicates larval failure. Cultivar preference has been considered evidence in favor of the preference-performance hypothesis, but this connection is not consistent across insect families [30,32,41,42]. Should female *H. testudinea* preferentially select cultivars for oviposition and if eggs and larvae have equal success across cultivars over the season, the cultivar preference of the female should correlate with cultivars exhibiting the most damage (e.g., high levels of secondary damage), consistent with the preference-performance hypothesis [25,26]. Results from our study provided only weak support (r = 0.4 and 0.2) for the preference-performance hypothesis. *Hoplocampa testudinea* females did not randomly oviposit across cultivar across multiple years, suggesting an orientation to cultivar based upon chemical cues [22], as observed for the apple blossom weevil [43] or possibly visual cues associated with the apple blossom [44]. However, the cultivar preference observed during oviposition in this study ranks the cultivars differently than is observed at harvest (see Table 1 and Table 2), further supporting the hypothesis that larval performance during development is contributing to observed results at harvest.

More than one *H. testudinea* egg per cluster and occasionally more than one egg/fruitlet was observed across all cultivars. In laboratory studies *H. testudinea* was observed to oviposit a single egg on a fruitlet and to produce an epideictic pheromone to deter conspecifics [45]. Other sawflies, such as the stem galling sawfly, *Euura lasiolepis* (Smith) (Hymenoptera: Tenthredinidae)*,* avoid ovipositing on arroyo willow shoots with natural or artificial oviposition scars [46] Roitberg [47] determined that adult *H. testudinea* had lower rates of oviposition on blossoms that were previously infested or artificially scarred to simulate oviposition. Our results demonstrate that populations of *H. testudinea* in Nova Scotia are not strongly repelled by con-specific oviposition. Avoidance of con-specifics could benefit *H. testudinea* by distributing the population throughout available hosts to avoid competition among offspring. With nearly 50% of clusters having no *H. testudinea* eggs it would seem that females seeking oviposition sites are not host limited and thus are more often choosing to oviposit on clusters with eggs. Multiple eggs within a cluster could lead to con-specific competition between larvae for fruitlets to develop, each instar requiring a fruitlet for food and shelter [16], or forcing a larvae to seek the next closest cluster and exposing them to predation or desiccation in the process. Predation and parasitism were factors we did not evaluate in this study but were considered to be low in this region as there are no reported instances of either in the literature. In a laboratory setting, *H. testudinea* larvae showed a preference for uninfested fruitlets when given the option between uninfested fruitlets and those with living/dead conspecific larvae inside. The cues larvae use in differentiating between host fruitlets is unknown [47]. Such discrimination could result in higher levels of secondary damage on a tree as larvae distribute themselves among available fruitlets within a cluster and between clusters.

**Figure 5:**
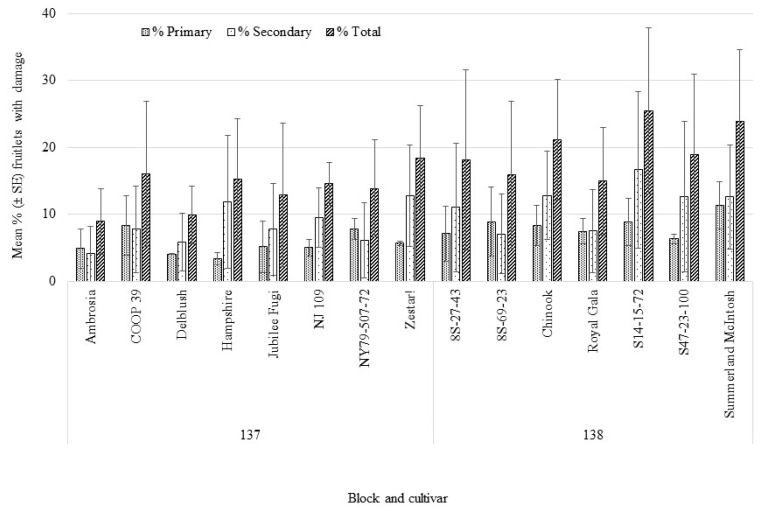
Mean (±SE) percentage of fruitlets showing primary, secondary and total damage from *Hoplocampa testudinea* observed in a bagged study of 15 cultivars in 2 blocks located at the Kentville Research and Development Centre in Nova Scotia over 3 years (2016, 2018 and 2019) and showing differences across cultivars. Primary: F_15,78_ = 1.07, *p* = 0.39, Secondary: F_15,78_ = 2.77, *p* = 0.002, Total: F_15,78_ = 2.85, *p* = 0.001.

The observed weak correlation between oviposition and secondary damage could be due to fruitlet chemistry resulting in differential survival of the larvae during development. Apple cultivar has been shown to influence fecundity and development of the two spotted spider mite [48]. In each year of our study, fruitlets from these cultivars exhibited significantly different physicochemical properties which may have had some impact on larval development. The combination of female preference during bloom (oviposition) and differential survival across cultivars may have resulted in the observed cultivar preference by *H. testudinea* at harvest and a differential ranking of cultivar throughout the growing season [22]. Development of larval *H testudinea* was correlated with chemical composition of the fruitlet. High levels of soluble solids, or sugars, had a negative impact on the success of 1st instar larvae, e.g., higher rates of primary damage, and on older instars, e.g., lower rates of secondary damage. Soluble solids, in general, increased during fruitlet development but to varying degrees. Sugars have been shown to be important for insect growth but are not considered essential for many insects [49]. Soluble sugar content influenced feeding behavior of the spruce budworm (*Choristoneura fumiferana* (Clem.)) [50] and diets with a high sugar content were found to increase pupal weight in *C. fumiferana* [51]. During development, *C. fumiferana* larvae receiving a high sugar diet during the later instar stages achieved high pupal weights while those on a low sugar diet later in their development had significantly lower mass. Soluble solids and acidity had very small effects on larval and survival weight in the Mediterranean fruit fly, *Ceratitis capitata* (Weidemann), but did have a significant impact on pupal weight [52]. Citrus with a high acid content (lemon at 6%) showed comparable larval survival to fruit with very low acid content (1.0%). Other compounds present in the fruit, not examined during this study, could also play a role in the results obtained. High phenol levels in wheat have been shown to deter aphids [53] and surface chemistry of the sweet potato influences preference in the sweet potato weevil [54]. Larval *H. testudinea* successfully surviving past the 1st instar (resulting in fruitlets with secondary damage) were negatively affected by fruitlet firmness. Cultivars with a low percentage of secondary damage had skin and flesh requiring high pressure to penetrate. *H. testudinea* larvae need to move between fruitlets and gain access by chewing through the skin. The increased time to penetrate the new fruitlet may leave the larvae vulnerable to predation or dessication. Fruit firmness is known to influence oviposition of *Drosophila suzukii* (Matsumura) where softer fruit are preferred over firmer fruit [55,56]. The full impact of *H. testudinea* on apple needs to be assessed by surveying fruitlets throughout their development and not just surveying apples at harvest. Fruitlets with primary damage represent less than 50% of the full impact of *H. testudinea* (Figure 5). The high variability associated with secondary damage is likely a result of environmental factors interacting with genotype to influence the acidity or sugar levels within the fruitlets as has been shown in cassava [57,58].

While cultivar preference in *H. testudinea* may appear to offer potential as a pest management tactic, there are some factors which may impact their use in this manner. The duration of the cropping cycle, i.e., up to or more than 10 years, and the likelihood that physiological changes over time will impact the observed cultivar preference should be considered when selecting cultivars. It has also been shown that cultivar preference can disappear when a single cultivar is planted [33] and cultivar preference is more apparent when a choice is provided [3]. While apple orchards typically represent a mixed cultivar situation, selecting and planting cultivars that are less preferred by *H. testudinea* could still create a preferred/non-preferred situation focusing the damage onto one or more of the cultivars within the orchard block. Cultivars least preferred by *H. testudinea*, or showing resistance, studied here showed negative impacts on the development of *H. testudinea* larvae (antibiosis) rather than preventing the insect from selecting the cultivar for oviposition (antixenosis). Newer cultivars, not examined in this study, could show greater evidence of antixenosis and this may improve the potential for using cultivars as a pest management strategy against *H. testudinea*.

Physicochemical characteristics of the fruitlets appear to play a role in the ability of larvae to survive and this varies across cultivar. Results from this study showed weak support for the preference-performance hypothesis and some evidence of antibiosis across cultivars during development. Larvae performed better on cultivars where fruitlets contained low acidity and high soluble solids during development. Cultivar preference in apple could be a multi-stage interaction where female choice during bloom is influenced by certain characteristics while larval development is affected by fruitlet chemistry which is affected by environment [59]. This creates a situation where direct correlation between female choice and offspring performance is less likely to be strong, or will be strong in certain years, but not others. Similarly, cultivars which show changes in ranking from year to year with respect to damage from *H. testudinea* could be more sensitive to variation in environmental conditions. Future studies should consider the response of the host under varying environmental conditions in addition to evaluation of the success, or failure, of the insect. Finally, for insect species which complete part of their life cycle inside the developing fruit and for crops which drop their damaged fruit, determination of susceptible and resistant cultivars requires evaluation throughout the growing season.

## Figures and Tables

**Table 1:** Percentage (±SE) of apples showing damage from *Hoplocampa testudinea* on cultivars from 2 blocks located at the Kentville Research and Development Centre, for all cultivars from 2010–2014 and for a subset of the cultivars from 2015–2019.

Block	Cultivar	2010–2014 ^a^	2015–2019 ^b^
137	8S-26-50	5.73 (1.92)	
	Ambrosia	2.12 (0.78)	4.41 (1.95)
	Autumn Gold	9.06 (1.92)	
	Chinook	10.8 (3.38)	
	COOP 29	2.71 (0.60)	
	COOP 39	8.57 (3.11)	6.37 (0.65)
	COR10T-17	5.65 (1.06)	
	Delblush	3.23 (1.18)	5.31 (2.24)
	Golden Delicious	8.69 (2.53)	
	Hampshire	7.76 (2.05)	5.75 (1.41)
	Jubilee Fugi	9.33 (1.79)	3.19 (0.76)
	NJ 109	2.26 (1.11)	6.74 (1.72)
	NJ 90	11.1 (6.16)	
	NY-65-707-19	4.39 (0.69)	
	NY-79-507-49	6.01 (1.37)	
	NY-79-507-72	10.6 (0.31)	8.07 (3.47)
	Pinova	4.07 (0.67)	
	Rogers’ McIntosh	6.02 (1.81)	
	Runkel	8.12 (1.89)	
	Zestar!	1.94 (0.80)	3.63 (2.01)
138	8NE-07-72	5.82 (1.40)	
	8S-26-50	7.50 (2.51)	
	8S-27-43	8.36 (1.78)	3.53 (1.29)
	8S-69-23	6.80 (0.94)	5.43 (1.48)
	Chinook	12.8 (2.68)	8.93 (1.40)
	Royal Gala	4.93 (1.29)	6.32 (1.01)
	S14-15-72	11.7 (2.31)	7.91 (2.69)
	S23-06-153	4.68 (0.61)	
	S43-43-79	6.29 (1.65)	
	S47-23-100	3.25 (1.13)	4.83 (2.29)
	Silken	5.23 (0.81)	
	Summerland McIntosh	5.58 (0.92)	5.91 (2.10)
	Zestar!	4.87 (3.16)	

^a^ Cultivar: Block 137: χ^2^ = 100.94, *p* < 0.0001, Block 138: χ^2^ = 72.91, *p* < 0.0001; ^b^ Cultivar: F_1,14_ = 1.23, *p* = 0.25.

**Table 2:** Mean percentage (±SE) of clusters with *Hoplocampa testudinea* eggs from 15 cultivars located in 2 blocks at Kentville Research and Development Centre from 2016–2019. Means within column and block with different letters significantly different (*p* < 0.05).

		Mean Percentage (±SE) of Clusters with Eggs				
Block	Cultivar	2016 *	2017	2018	2019	Across Years
137	Ambrosia	61.1 (8.4)	10.0 (4.1) b	5.6 (3.3) bc	12.2 (1.9) ab	22.2 (13.0) ab
	COOP 39	73.3 (6.9)	56.0 (7.5) a	70.0 (10.0) a	10.4 (4.7) ab	52.4 (14.5) a
	Delblush	34.0 (5.1)	14.0 (5.1) b	29.6 (10.7) bc	2.0 (2.0) bc	19.9 (7.3) b
	Hampshire	67.8 (15.8)	26.5 (5.7) a	46.0 (17.2) bc	16.5 (9.2) ab	39.2 (11.3) ab
	Jubilee Fugi	43.3 (13.3)	33.7 (10.4) ab	13.3 (8.8) bc	16.7 (6.7) ab	26.7 (7.1) ab
	NJ 109	54.0 (14.0)	38.0 (8.0) ab	40.0 (8.4) bc	24.9 (7.9) ab	39.2 (5.9) ab
	NY79-507-72	43.7 (14.6)	34.0 (6.8) ab	23.5 (7.2) ab	24.0 (7.5) ab	31.3 (4.8) ab
	Zestar!	56.0 (11.7)	22.0 (7.3) b	34.0 (5.1) bc	33.6 (5.4) a	36.4 (7.1) ab
138	8S-27-43	60.8 (10.8)	26.0 (9.3)	10.8 (7.8) c	26.0 (6.0) ab	30.9 (10.6) ab
	8S-69-23	21.5 (7.1)	48.0 (12.8)	36.2 (14.9) bc	22.0 (3.7) ab	31.9 (6.4) ab
	Chinook	65.6 (4.9)	46.0 (8.7)	55.2 (8.7) bc	10.4 (4.7) bc	44.3 (11.9) ab
	Royal Gala	62.5 (8.5)	33.3 (8.4)	33.6 (11.4) bc	16.7 (3.3) ab	36.5 (9.5) ab
	S14-15-72	59.7 (9.9)	39.0 (10.9)	42.5 (14.4) bc	47.3 (6.1) a	47.1 (4.5) ab
	S47-23-100	45.8 (10.1)	----	---	6.0 (6.0) bc	25.9 (19.9) ab
	Summerland McIntosh	36.4 (7.3)	58.0 (5.8)	64.0 (8.1) bc	36.0 (9.3) ab	48.6 (7.3) a

* **2016**: Block: F_1,58_ = 0.44, *p* = 0.51, Cultivar: F_13,58_ = 1.83, *p* = 0.059; **2017**: Block: F_1,56_ = 6.92, *p* = 0.01, Cultivar: F_12,56_ = 2.42, *p* = 0.013; **2018**: Block: F_1,48_ = 0.59, *p* = 0.44, Cultivar: F_12,48_ = 3.10, *p* = 0.002; **2019**: Block: F_1,55_ = 4.44, *p* = 0.03, Cultivar: F_13,55_ = 3.91, *p* < 0.001; **All years**: Block: F_1,261_ = 2.69, *p* = 0.10, Cultivar: F_13,261_ = 3.49, *p* < 0.0001.

**Table 3:** Mean number (±SE) of *Hoplocampa testudinea* eggs/cluster from 15 cultivars located in 2 blocks at Kentville Research and Development Centre from 2016–2019. Letters within year denote significant differences between cultivars, *p* < 0.05.

		Mean Number (±SE) Eggs/Cluster *				
Block	Cultivar	2016	2017	2018	2019	Across Years
137	Ambrosia	1.56 (0.2) ab	0.87 (0.3)	0.50 (0.3)	1.10 (0.1) abc	1.01 (0.1)
	COOP 39	1.78 (0.2) ab	1.31 (0.1)	1.21 (0.1)	0.70 (0.3) bc	1.25 (0.1)
	Delblush	1.36 (0.1) ab	0.80 (0.2)	1.63 (0.4)	0.40 (0.4) bc	1.03 (0.2)
	Hampshire	1.57 (0.2) ab	1.05 (0.1)	1.36 (0.4)	0.70 (0.3) bc	1.15 (0.1)
	Jubilee Fugi	1.47 (0.3) ab	0.97 (0.3)	0.77 (0.4)	1.00 (0.0) abc	1.04 (0.1)
	NJ 109	1.72 (0.3) ab	1.28 (0.2)	1.49 (0.2)	0.90 (0.2) bc	1.35 (0.1)
	NY79-507-72	1.81 (0.3) ab	1.22 (0.1)	1.50 (0.5)	1.39 (0.2) abc	1.48 (0.2)
	Zestar!	1.66 (0.2) ab	1.00 (0.3)	1.50 (0.2)	1.67 (0.2) ab	1.44 (0.1)
138	8S-27-43	2.21 (0.2) a	1.08 (0.3)	0.50 (0.3)	1.05 (0.1) bc	1.25 (0.2)
	8S-69-23	1.00 (0.3) b	1.68 (0.2)	1.45 (0.4)	1.10 (0.1) abc	1.31 (0.1)
	Chinook	1.91 (0.1) ab	1.47 (0.2)	1.76 (0.4)	0.70 (0.3) bc	1.49 (0.1)
	Royal Gala	1.94 (0.2) ab	1.17 (0.3)	1.46 (0.2)	1.00 (0.0) abc	1.39 (0.1)
	S14-15-72	1.59 (0.2) ab	1.52 (0.1)	1.20 (0.4)	1.81 (0.2) a	1.55 (0.1)
	S47-23-100	1.47 (0.2) ab	----	----	0.20 (0.2) bcd	0.83 (0.2)
	Summerland McIntosh	1.45 (0.2) ab	1.54 (0.2)	1.49 (0.2)	1.57 (0.2) ab	1.51 (0.1)

* **2016**: Block: F_1,58_ = 0.05, *p* = 0.82, Cultivar: F_13,58_ = 2.08, *p* = 0.03, **2017**: Block: F_1,56_ = 5.26, *p* = 0.03, Cultivar, F_12,58_ = 0.94, *p* = 0.52; **2018**: Block: F_1,48_ = 0.02, *p* = 0.96, Cultivar: F_12,48_ = 1.48, *p* = 0.16, **2019**: Block: F_1,55_ = 0.69, *p* = 0.41, Cultivar: F_13,55_ = 4.11, *p* < 0.0001, **All years**: Block: F_1,260_ = 2.71, *p* = 0.10, Cultivar: F_13,260_ = 1.94, *p* = 0.02.

**Table 4:** R^2^ and *p*-values for relationships between *Hoplocampa testudinea* eggs (mean percentage of clusters with eggs and mean numbers of eggs per cluster, *N* = 42), fruitlet chemistry and firmness and observed damage (mean percentage of fruit, *N* = 43) across 15 apple cultivars from 2 blocks over 3 years (2016, 2018 and 2019) located at the Kentville Research and Development Centre in Nova Scotia.

	**Total Damage**	**Primary Damage**	**Secondary Damage**
Percentage of clusters with eggs	**0.34, <0.0001**	0.01, 0.43	**0.40, <0.0001**
Total eggs/cluster	**0.35, <0.0001**	0.03, 0.29	**0.22, <0.001**
Percentage of eggs/cluster (on King)	0.09, 0.04	0.03, 0.27	0.07, 0.07
Percentage of eggs/cluster (on Laterals)	0.04, 0.22	0.007, 0.58	0.03, 0.27
Percentage of eggs/cluster (on King + Laterals)	**0.26, <0.0001**	0.03, 0.29	**0.22, <0.001**
% Soluble solids (at 10 mm)	0.02, 0.48	**0.22, 0.01**	0.13, 0.06
% Soluble solids (at 20 mm)	0.009, 0.63	0.13, 0.06	0.09, 0.13
% Soluble solids (at 30 mm)	**0.18, 0.005**	0.03, 0.27	**0.22, 0.002**
Change in soluble solids (10 to 20 mm)	0.007, 0.67	0.01, 0.54	<0.001, 0.99
Change in soluble solids (20 to 30 mm)	0.0008, 0.88	0.04, 0.76	0.02, 0.49
Acidity ^a^ (at 10 mm)	0.05, 0.26	**0.25, 0.008**	0.06, 0.22
Acidity (at 20 mm)	0.03, 0.35	0.003, 0.79	0.07, 0.15
Acidity (at 30 mm)	0.02, 0.36	0.001, 0.83	0.04, 0.20
Change in acidity (10 to 20 mm)	<0.001, 0.98	0.09, 0.12	**0.17, 0.03**
Change in acidity (20 to 30 mm)	0.02, 0.47	0.03, 0.35	<0.001, 0.90
Force (at 20 mm)	0.007, 0.67	0.11, 0.07	0.08, 0.13
Force (at 30 mm)	0.02, 0.42	0.02, 0.34	0.009, 0.54

^a^ measured by titrating 1 mL of juice in 50 mL of water using 0.1 N NaOH.

## Data Availability

Data available from the corresponding author upon request.

## Supplementary Materials

The following are available online at https://www.mdpi.com/article/10.3390/insects12090769/s1, Table S1. Mean (±SE) percentage of fruitlets bagged at petal fall and assessed in August of each year (2016, 2018 and 2019) from two blocks of apple trees located at Kentville Research and Development Centre in Nova Scotia which were healthy, showed damage from other insects (‘Other Damage’), failed to develop, had *Hoplocampa testudinea* damage and dropped from the tree (‘EAS drop’) or remained attached to the tree (‘EAS tree’) and combined damage (‘EAS total’). Figure S1: Mean (±SE) change in (A) percent soluble solids and (B) acidity of fruitlets during fruitlet development from 10 to 20 mm and from 20 to 30 mm in size. Fruitlets collected from 15 cultivars in two blocks located at the Kentville Research and Development Centre, Nova Scotia over 2017–2019. Note: “Delblush” cultivar was not included in this analysis due to lack of available fruitlets over all years and “S47-23-100” did not have sufficient fruitlets in 2017. Figure S2: (A) Mean (±SE) force required to break the skin of fruitlets collected when 20 and 30 mm in size and (B) change in force during development from 15 cultivars in 2 blocks located at the Kentville Research and Development Centre in Nova Scotia over 3 years (2017–2019).
