# Supplementary material for: A New Look at Cultivar Preference in Hoplocampa testudinea (Hymenoptera: Tenthredinidae) on Apple in the Annapolis Valley of Nova Scotia, Canada
Source: Insects. 2021 Aug 27;12(9):769. doi: 10.3390/insects12090769 (PMC8466372; doi:10.3390/insects12090769)

Supplemental Table S1: Mean (±SE) percentage of fruitlets bagged at petal fall and assessed in August of each year (2016, 2018 and 2019) from two blocks of apple trees located at Kentville Research and Development Centre in Nova Scotia which were healthy, showed damage from other insects (‘Other Damage’), failed to develop, had *Hoplocampa testudinea* damage and dropped from the tree (‘EAS drop’) or remained attached to the tree (‘EAS tree’) and combined damage (‘EAS total’).

			Mean (±SE) percentage of fruitlets (%)					
Year	Block	Cultivar	Healthy	Other Damage	Failed to Develop	EAS drop	EAS tree	EAS total
2016[a]	137	Ambrosia	47.7 (8.5)	10.3 (1.8)	25.1 (4.3)	10.5 (5.7)	6.3 (2.2)	16.8 (6.1)
		COOP 39	7.6 (1.8)	35.6 (12.7)	19.1 (11.9)	32.5 (5.2)	5.1 (5.1)	37.5 (4.5)
		Delblush	18.2 (5.8)	7.60 (1.4)	55.7 (5.3)	17.3 (1.5)	1.1 (0.4)	18.4 (1.3)
		Hampshire	24.2 (7.5)	8.3 (1.4)	34.2 (4.4)	28.8 (4.3)	4.5 (1.6)	33.3 (4.3)
		Jubilee Fugi	23.5 (5.6)	14.2 (1.8)	27.9 (6.5)	22.9 (4.9)	11.4 (2.7)	34.4 (3.3)
		NJ 109	20.7 (4.3)	10.1 (3.6)	48.9 (5.1)	18.8 (4.6)	1.4 (0.5)	20.2 (4.6)
		NY-79-507-72	30.9 (6.4)	12.1 (2.0)	35.0 (7.7)	16.6 (2.9)	5.3 (1.1)	21.9 (3.7)
		Zestar!	28.7 (4.4)	6.3 (1.2)	31.0 (3.5)	30.5 (3.2)	3.4 (1.2)	33.9 (3.4)
	138	8S-27-43	28.2 (5.6)	5.1 (3.1)	22.4 (6.9)	30.3 (8.2)	14.1 (4.4)	44.4 (11.4)
		8S-69-23	26.6 (9.2)	12.5 (11.6)	24.1 (9.4)	18.5 (6.0)	18.3 (0.9)	36.8 (7.0)
		Chinook	28.5 (5.4)	4.2 (1.9)	28.1 (6.6)	31.3 (4.1)	7.9 (1.1)	39.2 (3.4)
		Royal Gala	36.5 (5.5)	8.2 (2.8)	24.2 (2.8)	20.0 (7.7)	11.1 (2.3)	31.1 (7.0)
		S14-15-72	17.2 (2.1)	8.5 (1.8)	25.5 (0.6)	46.3 (3.3)	2.5 (1.7)	48.7 (2.1)
		S47-23-100	18.9 (3.6)	12.4 (3.7)	37.7 (4.4)	29.0 (5.4)	1.9 (0.7)	30.9 (4.9)
		Summerland McIntosh	11.6 (1.7)	16.2 (6.7)	27.2 (2.5)	39.0 (5.4)	5.9 (0.6)	44.9 (5.8)
2018[b]	137	Ambrosia	70.1 (17.4)	11.3 (1.3)	18.7 (18.7)	0.0 (0.0)	0.0 (0.0)	0.0 (0.0)
		COOP 39	30.4 (4.5)	2.7 (2.7)	64.3 (5.0)	0.0 (0.0)	2.7 (2.7)	2.7 (2.7)
		Delblush	28.0 (6.5)	0 (0.0)	65.5 (5.4)	0.0 (0.0)	6.5 (2.1)	6.5 (2.1)
		Hampshire	23.7 (4.5)	7.7 (4.5)	61.1 (0.6)	0.0 (0.0)	7.5 (2.3)	7.5 (2.3)
		Jubilee Fugi	46.8 (30.2)	19.4 (19.4)	31.2 (13.3)	0.0 (0.0)	2.5 (2.5)	2.5 (2.5)
		NJ 109	31.8 (8.4)	0.5 (0.5)	53.5 (9.0)	1.0 (1.0)	13.2 (4.1)	14.2 (4.8)
		NY-79-507-72	---	---	---	---	---	---
		Zestar!	48.4 (6.9)	16.3 (8.2)	22.6 (6.6)	5.2 (5.2)	7.4 (4.6)	12.6 (9.7)
	138	8S-27-43	37.5 (0.0)	0.0 (0.0)	62.5 (0.0)	0.0 (0.0)	0.0 (0.0)	0.0 (0.0)
		8S-69-23	41.6 (6.3)	5.6 (2.9)	52.8 (3.4)	0.0 (0.0)	0.0 (0.0)	0.0 (0.0)

		Chinook	49.5 (8.2)	0.0 (0.0)	37.6 (5.7)	0.0 (0.0)	12.9 (4.3)	12.9 (4.3)
		Royal Gala	66.4 (4.6)	11.5 (3.9)	15.8 (6.1)	0.7 (0.7)	5.5 (2.3)	6.2 (2.7)
		S14-15-72	30.1 (7.1)	5.9 (4.0)	57.8 (8.6)	0.0 (0.0)	6.4 (3.6)	6.4 (3.6)
		S47-23-100	---	---	---	---	---	---
		Summerland McIntosh	51.5 (7.9)	0.8 (0.8)	37.1 (5.1)	1.0 (1.0)	9.6 (4.5)	10.6 (4.6)
2019[c]	137	Ambrosia	37.7 (5.7)	12.1 (4.8)	40.2 (8.8)	0.0 (0.0)	10.0 (3.4)	10.0 (3.4)
		COOP 39	24.9 (3.7)	6.9 (3.1)	60.3 (6.9)	1.4 (1.0)	6.4 (2.4)	7.8 (3.1)
		Delblush	30.0 (3.1)	9.8 (4.7)	55.4 (3.8)	0.6 (0.6)	4.1 (2.9)	4.7 (2.8)
		Hampshire	43.1 (9.9)	23.2 (6.2)	28.8 (6.1)	0.9 (0.9)	3.9 (2.1)	4.8 (2.9)
		Jubilee Fugi	37.8 (3.1)	28.4 (7.5)	31.9 (7.1)	0.6 (0.6)	1.3 (1.3)	1.9 (1.9)
		NJ 109	17.9 (5.1)	6.7 (3.7)	66.2 (5.4)	2.3 (1.9)	6.9 (1.7)	9.2 (3.3)
		NY-79-507-72	25.5 (6.9)	3.2 (1.1)	64.7 (5.6)	0.4 (0.4)	6.2 (2.7)	6.6 (2.6)
		Zestar!	23.7 (3.9)	6.4 (1.8)	61.3 (7.6)	3.5 (2.4)	5.0 (2.6)	8.5 (4.9)
	138	8S-27-43	49.9 (4.3)	4.4 (2.1)	35.6 (5.1)	2.9 (0.7)	7.1 (1.4)	10.0 (1.9)
		8S-69-23	39.3 (5.7)	12.4 (2.3)	37.5 (7.1)	2.2 (0.7)	8.6 (3.4)	10.8 (3.8)
		Chinook	29.0 (2.2)	4.5 (1.9)	55.3 (6.5)	3.3 (2.8)	7.9 (3.9)	11.2 (6.6)
		Royal Gala	23.8 (1.7)	5.4 (2.1)	62.1 (1.6)	2.2 (0.6)	6.4 (1.6)	8.6 (1.9)
		S14-15-72	37.8 (11.9)	11.8 (2.6)	29.2 (8.9)	6.3 (1.3)	14.9 (4.4)	21.2 (3.9)
		S47-23-100	30.0 (4.5)	2.9 (1.4)	60.1 (3.3)	1.4 (0.6)	5.5 (1.3)	6.9 (1.9)
		Summerland McIntosh	27.9 (4.7)	6.2 (1.1)	49.7 (3.3)	3.7 (1.0)	12.4 (5.0)	16.1 (5.5)

[a]Healthy: $F_{14,54} = 3.52$, $P < 0.0001$, Other Damage: $F_{14,54} = 2.59$, $P = 0.006$, Failed to Develop: $F_{14,54} = 2.99$, $P = 0.002$, EAS drop: $F_{14,54} = 2.96$, $P = 0.002$, EAS tree: $F_{14,54} = 4.29$, $P < 0.0001$, EAS total:$F_{14,54} = 3.31$, $P < 0.0001$

[b]Healthy: $F_{12,34} = 2.92$, $P = 0.007$, Other Damage: $F_{12,34} = 2.24$, $P = 0.03$, Failed to Develop: $F_{12,34} = 5.81$, $P < 0.0001$, EAS drop: $F_{12,34} = 1.01$, $P = 0.46$, EAS tree: $F_{14,55} = 1.22$, $P = 0.31$, EAS total: $F_{12,34} = 1.39$, $P = 0.22$

[c]Healthy: $F_{14,55} = 2.13$, $P = 0.02$, Other Damage: $F_{14,55} = 4.22$, $P < 0.0001$, Failed to Develop: $F_{14,55} = 4.25$, $P < 0.0001$, EAS drop: $F_{14,55} = 1.48$, $P = 0.15$, EAS tree: $F_{14,55} = 1.19$, $P = 0.31$, EAS total: $F_{14,55} = 1.42$, $P = 0.17$

Supplemental Figure S1: Mean (± SE) change in A) percent soluble solids and B) acidity of fruitlets during fruitlet development from 10 to 20 mm and from 20 to 30 mm in size. Fruitlets collected from 15 cultivars in two blocks located at the Kentville Research and Development Centre, Nova Scotia over 2017-2019. Note: "Delblush" cultivar was not included in this analysis due to lack of available fruitlets over all years and "S47-23-100" did not have sufficient fruitlets in 2017.

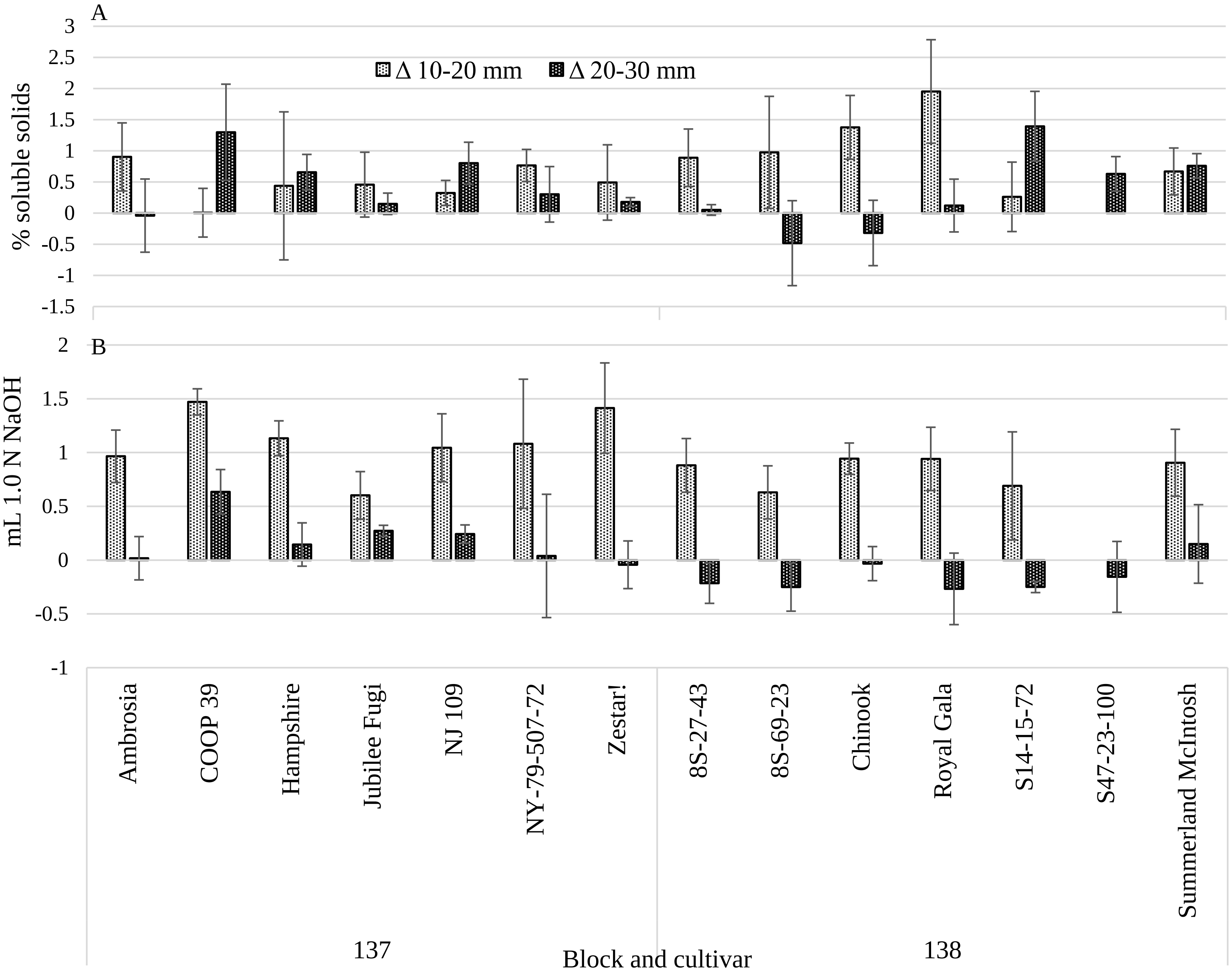

Supplemental Figure S2: (A) Mean (±SE) force required to break the skin of fruitlets collected when 20 and 30 mm in size and (B) change in force during development from 15 cultivars in 2 blocks located at the Kentville Research and Development Centre in Nova Scotia over 3 years (2017-2019).

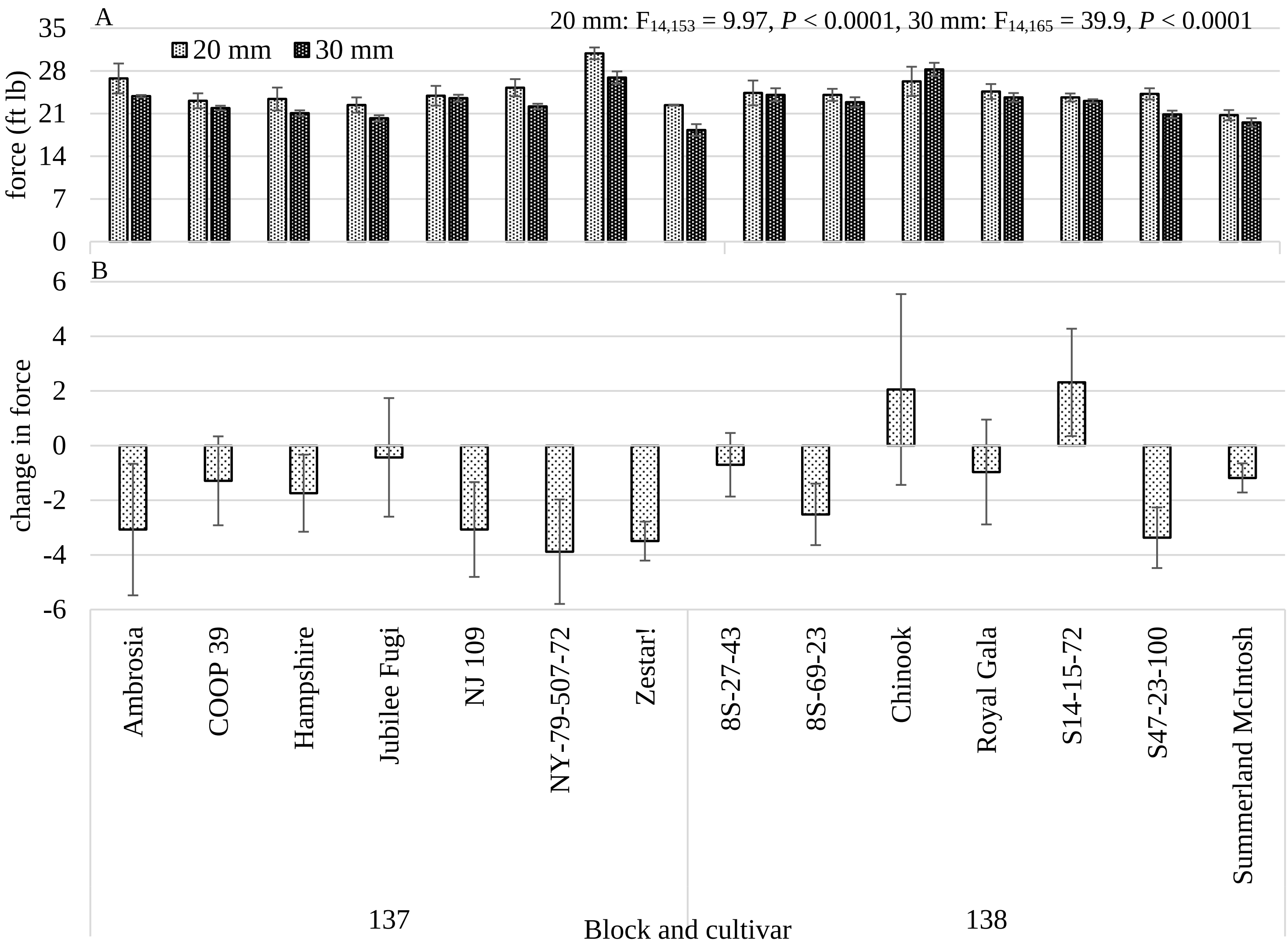

Supplement: Supplementary file 1 [file insects-12-00769-s001.zip › insects-1331017-supplementary.pdf]
